# Association between Administration of Antithrombotics and Intraperitoneal Hemorrhage in Patients Undergoing Percutaneous Interventions for Liver Diseases

**DOI:** 10.3390/jcm10112527

**Published:** 2021-06-07

**Authors:** Akira Asai, Keisuke Yokohama, Hideko Ohama, Yusuke Tsuchimoto, Shinya Fukunishi, Kazuhide Higuchi

**Affiliations:** 2nd Department of Internal Medicine, Osaka Medical College, Takatsuki 5698686, Japan; hammer_0906@yahoo.co.jp (K.Y.); in2152@osaka-med.ac.jp (H.O.); shibaraku6960@yahoo.co.jp (Y.T.); in2104@osaka-med.ac.jp (S.F.); higuchi@osaka-med.ac.jp (K.H.)

**Keywords:** intraperitoneal hemorrhage, percutaneous interventions, antithrombotics, liver disease

## Abstract

Currently, percutaneous interventions are essential for diagnosis and treatment of liver diseases. The most frequent complication of percutaneous interventions is intraperitoneal hemorrhage. Recently, the number of patients with liver diseases on antithrombotics has been increasing. This retrospective cohort study aimed to evaluate the risk factors for intraperitoneal hemorrhage in patients after percutaneous interventions for liver diseases. This study included 1025 patients who underwent percutaneous interventions for liver diseases from April 2015 to March 2020. All interventions were performed using an ultrasound-guided approach. The influence of antithrombotic drug administration in patients, who underwent percutaneous interventions according to the guidelines for the American Association for the Study of Liver Disease, was evaluated. Intraperitoneal hemorrhage after percutaneous interventions was detected by computed tomography. Intraperitoneal hemorrhage occurred in nine patients (0.88%); however, these adverse events were not severe. We compared clinical characteristics between the patients with and without intraperitoneal hemorrhage. Although, there was no difference based on the administration of antithrombotics (*p* = 0.1961), seven of nine patients who showed intraperitoneal hemorrhage received percutaneous treatments (radio frequency ablation or microwave ablation). Therefore, we divided patients who underwent treatments and liver biopsy and then investigated the influence of antithrombotics on the intraperitoneal hemorrhage. After propensity score matching in each patient group, the administration of antithrombotics was not identified as a risk factor for hemorrhage in patients who underwent interventional treatments and patients who underwent liver biopsy. When the antithrombotics were discontinued, according to the guidelines, it may not increase the risk factor for hemorrhage in patients of liver disease who underwent percutaneous interventions.

## 1. Introduction

There were 123 million prevalent cases of cirrhosis in 2017 worldwide [1]. Percutaneous interventions for liver diseases are currently essential for both diagnosis and treatment [2,3,4]. Although, these interventions were previously performed blindly or guided by computed tomography (CT), recently, most of them have been ultrasound guided [5]. Percutaneous interventions for diagnosis are mostly performed by liver biopsy. Liver biopsy is performed for histopathological interpretation when information for diagnosis, management, or prognosis is not available from non-invasive techniques. Percutaneous interventions for the treatment of liver diseases are mainly performed for liver tumors including hepatocellular carcinoma and metastatic liver tumor. In addition, radio frequency ablation (RFA) and microwave ablation (MWA) are used in these treatments. These are interventions that use ultrasound image guidance to place a needle through the skin into liver tumors. Worldwide, liver cancer was the fifth most common cancer in 2017 with an estimated 953,000 new cases [6]. However, complications of percutaneous interventions, such as hemorrhage, liver abscess, hepatic injury, extrahepatic organ injury, tumor progression, and thermal injuries to the skin, have been reported. The most frequent complication of these interventions is intraperitoneal bleeding (0.2%) [7,8,9]. Most cases of intraperitoneal hemorrhage can be managed by observation and hemostatic agents; however, some cases have been reported to require blood transfusion or become fatal [10,11].

Recently, patients with chronic liver diseases who are administered antithrombotic drugs, such as antiplatelet and anticoagulant drugs, have been increasing [12]. There are two reasons for this: These patients are aging and sometimes have multiorgan diseases for which antithrombotic agents are required [13]. The other reason might be that the ratio of non-alcoholic steatohepatitis (NASH), among other causes of chronic liver diseases, in these patients is increasing [14]. Non-alcoholic steatohepatitis is associated with extrahepatic manifestations such as cardiovascular disease, hypertension, and hyperlipidemia [15,16]. Therefore, these patients need to be administered single or dual antithrombotic therapy. However, in many patients with chronic liver disease, there are inherently abnormalities in hematological parameters with disturbances in both thrombolysis and coagulation. Usually, interventions are performed according to the guidelines for liver biopsy by the American Association for the Study of Liver Disease (AASLD) and the British Society of Gastroenterology (BSG) [17,18]. In these guidelines for liver biopsy, discontinuation of antithrombotic therapy is recommended for patients before interventions. However, the evidence for the particular timeframe for the discontinuation of antithrombotics stated in the guidelines is weak. Moreover, there are no specific guidelines for percutaneous treatments of liver tumors. In addition, no recommendations for using antithrombotic drugs before other percutaneous procedures are available. There are concerns that the discontinuation of antithrombotic drugs may worsen the disease [19,20]. In this retrospective cohort study of patients who received percutaneous interventions for liver diseases, we studied the incidence rates of intraperitoneal hemorrhage in patients after percutaneous interventions who administrated the antithrombotics according to the guideline.

## 2. Materials and Methods

### 2.1. Ethics and Informed Consent

All procedures performed in this study were in accordance with the ethical standards of the institution and ethical guidelines for medical and human subjects in Japan and with the 1964 Helsinki Declaration and its later amendments. This retrospective study was approved by the Institutional Review Board of Osaka Medical College (IRB approval number: 2020-070). Informed consent was obtained in the form of an opt-out, and patients who rejected to participate were excluded from the study. Ethical approval was obtained from the Ethical and Scientific Committee of the Osaka Medical College.

### 2.2. Study Design and Participants

At Osaka Medical College Hospital, 1025 patients who underwent percutaneous interventions for liver diseases (liver biopsy and percutaneous ablation such as RFA and MWA) from April 2015 to March 2020 were enrolled in this study (Table 1). All interventions were performed under an ultrasound-guided approach using an XALIO ultrasound system or APLIO i800 (Canon Medical Systems Corporation, Tochigi, Japan). Of these, 297 patients underwent a non-targeted liver biopsy, and 181 patients underwent a targeted biopsy for liver tumors. A total of 547 patients who were diagnosed with liver tumors (522 hepatocellular carcinomas and 25 metastatic other cancers) underwent percutaneous interventions (RFA or MWA). Additionally, 499 patients underwent RFA and 58 patients underwent MWA for other indications. We retrospectively collected the following patient clinical and demographic data: age, sex, etiology of liver disease, administration of antithrombotic drugs, laboratory data, and information about tumors (i.e., tumor size, number of tumors, and distance from the liver surface to tumor). We used a 14 G side-cutting needle for liver biopsies and a 21 G needle for targeted biopsies for liver tumors. Regarding percutaneous interventions for treatment, we used 17 G needles (cool-tip™ RF needle) for RFA and 14 G needles (Thermosphere Technology with Emprint™ Long Percutaneous Antenna) for MWA. When patients administered with antithrombotic drugs were scheduled to undergo percutaneous interventions, antiplatelet drugs were paused 5–7 days before the intervention, according to AASLD guidelines [17]. In addition, warfarin was discontinued 5 days before intervention with point-of-care testing before the intervention to ensure adequate reversal. Only 28 patients who were administered warfarin were switched to low-molecular-weight heparin via subcutaneous injection, and this injection was discontinued 12 h before the respective interventions. Abdominal CT was used for the detection of intraperitoneal hemorrhage after percutaneous intervention until seven days after the intervention.

### 2.3. Statistical Analysis

Study variables are presented as mean ± standard deviation for continuous variables and number (%) for categorical variables. Clinical laboratory values were not normally distributed. Therefore, the Mann-Whitney U test was used to analyze proportional scales. Fisher’s exact test was used to analyze nominal scales. All recorded *p*-values were two-sided, and a value of *p* < 0.05 was considered statistically significant. All analyses were performed using JMP software, version 13 (SAS Institute Inc., Cary, NC, USA). In the analysis of the prevalence of intraperitoneal hemorrhage in patients who were or were not administered antithrombotic drugs, propensity score matching was performed by using the variables of the logistic regression model. Propensity score matching was performed for age, sex, liver cirrhosis, and environments of tumors.

## 3. Results

### 3.1. Patient Characteristics

Baseline clinical characteristics of 1025 patients who underwent percutaneous interventions (liver biopsy and treatment of liver tumor such as RFA and MWA) are shown in Table 1. Antithrombotic drugs were administered to 163 patients (15.9%) who underwent percutaneous interventions. A total of 138 patients (13.4%) were treated with monotherapy of antithrombotic drugs, and 25 patients (2.4%) received combination therapy. The most common antithrombotic therapy was antiplatelet drugs (102 patients (10.0%)), and only 47 patients (4.6%) were treated with anticoagulant drugs. One hundred and sixty patients discontinued these antithrombotic drugs before percutaneous interventions in accordance with the AASLD guidelines for liver biopsy. Intraperitoneal hemorrhage occurred in nine patients (0.88%), and hemoglobin levels decreased by 1.77 ± 1.02 g/dL after these hemorrhages. Although blood transfusion was required in four patients, invasive hemostatic operation was not required in this study. When patients discontinued antithrombotic therapies before interventions, three-point major adverse cardiovascular events (i.e., cardiovascular death, nonfatal myocardial infarction, and nonfatal stroke) were not detected in these patients until discharge to home. Twenty-five patients treated with combination therapy discontinued these drugs according to the AASLD guidelines for liver biopsy, and there was no intraperitoneal hemorrhage after interventions in these patients. In conclusion, intraperitoneal hemorrhage occurred in only nine patients (0.88%), and these adverse events were not severe.

### 3.2. Risk Factors of Intraperitoneal Hemorrhage in Patients after Percutaneous Interventions

Next, we evaluated which factors were associated with intraperitoneal hemorrhage after percutaneous interventions (Table 2). Baseline clinical characteristics between the patients with intraperitoneal hemorrhage (positive group) and those without (negative group) were compared. The ratio of patients with liver cirrhosis and the etiology of liver disease were not significantly different between the two groups. The number of patients who underwent percutaneous treatments for liver tumor in the positive group was similar to those in the negative group. In addition, the number of patients who were administered antithrombotic drugs in the positive group was similar to those in the negative group.

We assessed the clinical characteristics of the nine patients with intraperitoneal hemorrhage after percutaneous interventions (Table 3). Seven of these nine patients underwent percutaneous treatments. Intraperitoneal hemorrhage was observed in the treatment of tumors of 2 cm or less and was also observed in those with a distance of 4 cm or more from the liver surface. In four patients, no abnormality of hematological parameters with disturbance of thrombolysis or coagulation was detected. Furthermore, three patients were administered antithrombotic drugs, and all of them responded according to the guidelines for antithrombin drugs.

### 3.3. Influence of Antithrombotic Drugs on Intraperitoneal Hemorrhage in Patients Who Underwent Percutaneous Interventions

The influence of antithrombotic drug administration in patients who underwent percutaneous interventions was evaluated. Since we considered that different interventions may affect intraperitoneal hemorrhage, we first analyzed the influence of antithrombic drug administration in patients who underwent percutaneous treatments. We divided these patients into those with and without antithrombotic drugs. Because of some confounding factors in the clinical background in the two groups of patients, we performed propensity score matching for some factors between these two groups of patients (Table 4). In each group, 100 patients were matched, and most of them who were treated with antithrombotic drugs discontinued these drugs according to the guidelines before percutaneous treatments. There was no difference in liver function and hematological parameters between the two groups. However, serum albumin was lower in patients with antithrombotic drugs (*p* = 0.0324). Two patients treated with antithrombotic drugs observed intraperitoneal hemorrhage after interventions. This adverse event was detected in one patient without antithrombotic drugs and there was no significant difference between the two groups (*p* = 0.5570). Second, we analyzed the influence of antithrombic drug administration in patients who underwent liver biopsy (Table 5). We also performed propensity score matching for some factors. A total of 58 patients were matched in the two groups, and all patients with antithrombotic drugs discontinued these drugs according to the guidelines before liver biopsy. There was no hemorrhage in both groups of patients.

Based on these results, intraperitoneal hemorrhage was observed in nine of 1025 patients who underwent percutaneous interventions. Moreover, the administration of antithrombotic drugs was not an increased risk factor for this hemorrhage in patients who underwent percutaneous interventions.

## 4. Discussion

There are two major guidelines about liver biopsy, AASLD and BSG. Although there is no difference in the particular timeframe for the discontinuation of antithrombotics stated in both guidelines, the evidences for the timeframe for discontinuation are weak. We performed interventions according to the guidelines of the AASLD for liver biopsy [17], and the administration of antithrombotic drugs was not found to be a risk factor for intraperitoneal hemorrhage in patients who underwent percutaneous interventions in this study. Percutaneous interventions for liver disease was less invasive rather than hepatectomy and, thus, it may be possible to shorten the timeframe of discontinuation. In this study, there were three patients in who were administered aspirin and also underwent liver biopsy, and no hemorrhage was observed. Furthermore, it was reported that the discontinuation of antithrombotics in procedures is a risk factor of thromboembolic complications. As per the guidelines for gastroenterological endoscopy, in 1137 patients undergoing antithrombotic treatment, thromboembolic complications due to the fact of warfarin withdrawal resulted in strokes in 12 patients [21,22]. Further research about the timeframe of discontinuation of antithrombotics in percutaneous interventions for liver diseases is needed. 

It has been reported that hemorrhage was detected in 0.4–0.5% of patients who underwent abdominal interventions [7,8,9]. In this study, intraperitoneal hemorrhage occurred in nine patients who underwent percutaneous interventions (0.88%). However, in previous reports, intraperitoneal hemorrhage was defined as the requirement for blood transfusion after interventions or reducing serum hemoglobin by more than 3 g/dL before interventions. In this study, intraperitoneal hemorrhage was defined based on diagnosis by CT until 7 days after interventions. In our study, the patients with intraperitoneal hemorrhage included even milder hemorrhage than in other studies, suggesting an increase in the frequency of these complications. In all nine patients with intraperitoneal hemorrhage, a reduction in serum hemoglobin level by 3 g/dL or more was not observed, and there were no abdominal symptoms. Moreover, five of these patients were conservatively followed up with hemostatic agents alone, and only four patients required blood transfusions. It has been reported that the posterior intercostal artery could be injured by these interventions [23]. There are some reports that hemorrhage could not be controlled by blood transfusion and patients died after these interventions [10,24,25]. Therefore, these interventions should be performed carefully.

In this study, the patients who underwent RFA or MWA had more frequent adverse events of intraperitoneal hemorrhage than those who underwent liver biopsy. Various factors, such as the gauge of the needle and number of punctures, may be involved in intraperitoneal hemorrhage after percutaneous interventions. Especially, it has been reported that localization of targeted tumors is one of the risk factors for intraperitoneal hemorrhage in patients with liver tumors after RFA [26], and a high frequency of hemorrhage occurred in cases where the tumors existed near the liver surface [7]. In this study, the gauge of needle, the number of punctures, and the distance from the targeted tumors to the liver surface did not differ in patients with or without hemorrhage.

This study had several limitations. It had all limitations inherent to retrospective studies: prone to selection bias and subject to confounding. However, we performed propensity score matching for some factors between these the two groups of patients to avoid the effect of confounding variables on the results. Single center study was another limitation that impacts on generalizability; however, in surgical interventional studies it offers the advantage of skills of surgeon being constant. The small sample size is another limitation of the study that could have reduced the power of the study and increased the margin of error.

## 5. Conclusions

The administration of antithrombotic drugs according to the guidelines may not be a risk factor for intraperitoneal hemorrhage in patients who underwent percutaneous interventions.

## Figures and Tables

**Table 1:** Baseline characteristics of the patients who underwent percutaneous interventions.

	(*n* = 1025)
Sex (male/female)	648/377
Age (years), median (range)	73.0 (16–90)
Patients with liver cirrhosis	596
Patients treated with percutaneous treatments (RFA and MWA)	547
Etiology of liver disease (HCV/HBV/NASH/ALD/other)	408/97/85/136/299
Patients who were administered antithrombotic drugs	163
(monotherapy/combination therapy)	138/25
Patients administered antiplatelet drugs	102
Patients administered anticoagulant drugs	47
Patients administered another antithrombotic drug	31
Patients who discontinued antithrombotic drugs before interventions	160
Baseline hemoglobin (g/dL)	12.60 ± 1.81
Platelet count (×10^4^/μL)	15.99 ± 8.30
Albumin (g/mL)	3.72 ± 0.55
Total bilirubin (mg/dL)	0.85 ± 1.09
PT (%)	89.54 ± 15.68
APTT (seconds)	32.94 ± 4.89
Child-Pugh score	5.59 ± 0.94
Patients with intraperitoneal hemorrhage after interventions	9 (0.88%)
Decrease in value of hemoglobin (g/dL)	1.77 ± 1.02

Abbreviations: RFA, radiofrequency ablation; MWA, microwave ablation; HCV, hepatitis C virus; HBV, hepatitis B virus; NASH, non-alcoholic steatohepatitis; ALD, alcoholic liver disease; PT, prothrombin time; APTT, activated partial thromboplastin time.

**Table 2:** Characteristics of patients with intraperitoneal hemorrhage or without after percutaneous interventions.

	Positive Group	Negative Group	*p-*Value
	(*n* = 9)	(*n* = 1016)	
Sex (male/female)	5/4	643/373	0.6361
Age (years), median (range)	73 (48–87)	73 (16–90)	0.5093
Patients with liver cirrhosis	6	590	0.5893
Patients treated with percutaneous treatments (RFA and MWA)	7	540	0.1271
Etiology of liver disease (HCV/HBV/NASH/ALD/other)	2/3/1/1/2	406/93/84/135/297	0.3519
Patients who were administered antithrombotic drugs	3	160	0.1961
(monotherapy/combination therapy)	3/0	135/25	0.2618
Patients administered antiplatelet drugs	2	100	0.2782
Patients administered anticoagulant drugs	1	46	0.4221
Patients administered another antithrombotic drug	0	31	0.4562
Patients who discontinued antithrombotic drugs	3	157	0.7373
Platelet count (×10^4^/μL)	13.54 ± 7.35	16.02 ± 8.31	0.3244
Albumin (g/mL)	3.72 ± 0.28	3.72 ± 0.55	0.9744
Total bilirubin (mg/dL)	0.74 ± 0.53	0.85 ± 1.09	0.7075
PT (%)	81.78 ± 19.24	89.01 ± 16.04	0.1683
APTT (seconds)	31.16 ± 2.30	32.99 ± 4.94	0.3112
Child-Pugh score	5.56 ± 0.88	5.59 ± 0.94	0.9029

Abbreviations: RFA, radiofrequency ablation; MWA, microwave ablation; HCV, hepatitis C virus; HBV, hepatitis B virus; NASH, non-alcoholic steatohepatitis; ALD, alcoholic liver disease; PT, prothrombin time; APTT, activated partial thromboplastin time.

**Table 3:** Characteristics of nine patients with intraperitoneal hemorrhage.

	Age	Sex	Interventions	Maximum Size of Tumors (mm)	Number of Targets	Distance from Target to Surface (mm)	Etiology	Cirrhosis	Platelets (×10^4^ mL)	PT (%)	APTT (sec)	Albumin (g/mL)	Bilirubin (mg/dL)	Child-Pugh Score	Decreasing of Hemoglobin (g/dL)	Antithrombotic Drugs
1	73	M	RFA	19	2	3	HBV	+	3.8	83	N/D	3.6	1.1	5	1.6	−
2	63	M	RFA	15	1	2	HBV	+	4.7	69	N/D	3.5	1.5	7	0.4	−
3	87	F	RFA	35	1	52	HCV	+	22.6	39	33.8	3.9	0.5	7	2.6	+
4	77	M	MWA	18	3	2	HBV	−	15.2	101	N/D	4.1	0.2	5	2.9	−
5	78	F	RFA	19	2	4	HCV	+	18.7	75	N/D	3.6	0.7	5	2	+
6	82	M	RFA	22	1	42	NASH	+	9.5	85	29.9	3.2	0.3	6	2.2	+
7	73	M	RFA	15	4	11	ALD	+	7.2	91	31.3	3.8	1.6	5	1.1	−
8	70	F	Biopsy	-	-	-	PBC	−	21.7	101	32.8	3.8	0.5	5	2.9	−
9	48	F	Biopsy (Targeted)	12	1	17	Hemangioma	−	18.5	91	28.0	4.0	0.3	5	0.2	−

**Table 4:** Characteristics of patients with antithrombotic drugs and without who received percutaneous treatments (RFA and MWA).

	Patients with Antithrombotic Drugs	Patients without Antithrombotic Drugs	*p-*Value
	(*n* = 100)	(*n* = 100)	
Gender (male/female)	80/20	81/19	0.8583
Age (years), median (range)	76 (56–90)	76 (56–87)	0.7833
Patients with liver cirrhosis	87	84	0.5465
Etiology of liver disease (HCV/HBV/NAFLD/ALD/other)	48/16/4/24/8	57/14/2/21/6	0.7225
Intraperitoneal hemorrhage after percutaneous interventions	2	1	0.557
Platelet count (×10^4^/μL)	11.60 ± 4.20	12.58 ± 4.34	0.1063
Albumin (g/mL)	3.55 ± 0.54	3.71 ± 0.51	0.0324
Total bilirubin (mg/dL)	0.76 ± 0.40	0.83 ± 0.40	0.2544
PT (%)	84.95 ± 15.60	86.59 ± 13.06	0.4211
APTT (seconds)	33.05 ± 4.24	32.36 ± 2.07	0.5363
Child-Pugh score	5.71 ± 0.94	5.59 ± 0.88	0.3505
Number of target tumors for treatment	1.3 ± 0.6	1.2 ± 0.5	0.2619
Maximum size of target tumors (cm)	1.71 ± 0.71	1.67 ± 0.69	0.6936
Minimum distance from liver surface to target tumors (cm)	2.29 ± 1.98	2.29 ± 1.80	0.9873

Abbreviations: RFA, radiofrequency ablation; MWA, microwave ablation; HCV, hepatitis C virus; HBV, hepatitis B virus; NASH, non-alcoholic steatohepatitis; ALD, alcoholic liver disease; PT, prothrombin time; APTT, activated partial thromboplastin time.

**Table 5:** Characteristics of patients with antithrombotic drugs and without who received liver biopsy.

	Patients with Antithrombotic Drugs	Patients without Antithrombotic Drugs	*p-*Value
	(*n* = 58)	(*n* = 58)	
Gender (male/female)	41/17	41/17	1
Age (years), median (range)	75 (29–88)	74 (27–89)	0.9937
Patients with liver cirrhosis	20	17	0.5499
Etiology of liver disease (HCV/HBV/NAFLD/ALD/other)	19/6/4/6/23	17/2/4/4/31	0.4343
Intraperitoneal hemorrhage after percutaneous liver biopsy	0	0	
Platelet count (×10^4^/μL)	20.76 ± 8.09	21.10 ± 10.72	0.8492
Albumin (g/mL)	3.83 ± 0.51	3.78 ± 0.60	0.6422
Total bilirubin (mg/dL)	0.72 ± 0.53	0.84 ± 1.00	0.4045
PT (%)	88.86 ± 17.91	93.62 ± 17.03	0.1454
APTT (seconds)	36.37 ± 7.99	31.61 ± 2.65	0.0377
Child-Pugh score	5.48 ± 0.78	5.60 ± 1.20	0.5213

Abbreviations: RFA, radiofrequency ablation; MWA, microwave ablation; HCV, hepatitis C virus; HBV, hepatitis B virus; NASH, non-alcoholic steatohepatitis; ALD, alcoholic liver disease; PT, prothrombin time; APTT, activated partial thromboplastin time.

## Data Availability

The data presented in this study are available on request from the corresponding author.
